# Case Report: A Novel *CXCR4* Mutation in a Chinese Child With Kawasaki Disease Causing WHIM Syndrome

**DOI:** 10.3389/fimmu.2022.857527

**Published:** 2022-04-13

**Authors:** Xiaopeng Ma, Yaping Wang, Peng Wu, Meiyun Kang, Yue Hong, Yao Xue, Chuqin Chen, Huimin Li, Yongjun Fang

**Affiliations:** ^1^ Department of Hematology and Oncology, Children’s Hospital of Nanjing Medical University, Nanjing, China; ^2^ Key Laboratory of Hematology, Nanjing Medical University, Nanjing, China

**Keywords:** WHIM syndrome, Kawasaki disease, *CXCR4*, genetics, novel mutation

## Abstract

WHIM syndrome, an extremely rare congenital disease with combined immunodeficiency, is mainly caused by heterozygous gain-of-function mutation in the *CXCR4* gene. There have been no previous case reports of WHIM syndrome with Kawasaki disease. We herein report a case of a boy who developed Kawasaki disease at the age of 1 year. After treatment, the number of neutrophils in his peripheral blood decreased continuously. His medical history revealed that he had been suffering from leukopenia, neutropenia and low immunoglobulin since birth, and his neutrophils could return to the normal level in the presence of infection or inflammation. Clinical targeted gene sequencing of 91 genes associated with granulocyte-related disease revealed that the patient had a novel heterozygous NM_003467; c.1032_1033delTG;p.(E345Vfs*12) variant in exon 2 of *CXCR4* gene. Family verification analysis by Sanger sequencing showed that his father also had heterozygous variation at this site, while other family members did not. The computer prediction software indicated that the variation had a high pathogenicity. The computational structure analysis of the mutant revealed significant structural and functional changes in the CXCR4 protein. It should be noted that when unexplained persistent neutropenia with low immunoglobulin occurs after birth, especially when there is a family history of neutropenia, immunodeficiency should be investigated with genetic testing.

## Introduction

**Figure 1:**
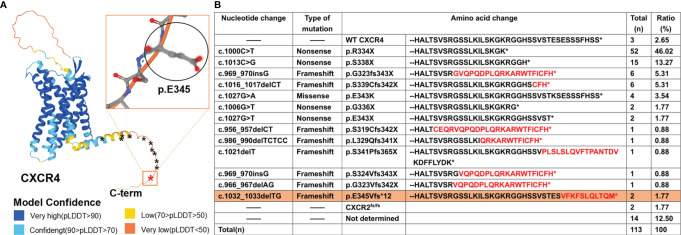
WHIM syndrome caused by mutations in C-terminal of CXCR4 and other causes. **(A)** Three-dimensional (3D) structure of CXCR4, predicted by using AlphaFold developed by DeepMind and EMBL-EBI, is shown on the left with stars (*) demarcating locations of WHIM mutations. The novel mutation identified in this study is marked in red, with the rectangular box showing its 3D structure, and the previously reported mutations in the *CXCR4* gene are marked in black. AlphaFold produces a per-residue confidence score (pLDDT) between 0 and 100. Some regions below 50 pLDDT is unstructured in isolation, which is a reasonably strong predictor of disorders. **(B)** Summary of 111 reported cases in the WHIM syndrome spectrum literature and two novel cases (in this study). Neo-sequence imposed by frame-shift mutations is in red. The novel mutation is highlighted with orange background.

WHIM syndrome, an extremely rare congenital disease with combined immunodeficiency, is caused by autosomal dominant inheritance (1). In 1964, Krill and his colleagues first described this disease, which was then named by the acronym of its four main clinical manifestations (warts, hypogammaglobulinemia, infections, and myelokathexis) and included in the online Mendelian human inheritance in 1990 (2, 3). In 2003, George Diaz first confirmed that this disease is caused by heterozygous mutations of *CXCR4* gene (4). C-X-C chemokine receptor type 4 (CXCR4), encoded by the *CXCR4* gene located on human chromosome 2q22.1, is a member of chemokine receptor family and its ligand is bone marrow stromal cell-derived factor (SDF-1, CXCL12). It is widely expressed in a variety of nucleated cells and plays an important role in many cellular functions, including hematopoiesis, immune monitoring, tumor growth and metastasis (5–7). Like all G protein coupled receptors (GPCRs), CXCR4 consists of 7 transmembrane helices, an extracellular N-terminal domain and an intracellular C-terminal domain (8). In addition to participating in intracellular signal transduction, its C-terminal also acts as a mutation-prone site, as confirmed by in silico analyses on the three-dimensional (3D) structure of CXCR4 protein predicted by AlphaFold from its amino acid sequence (**Figure 1A**) (9, 10). As of October 2021, a total of 113 cases have been reported (including 2 reported in this article) and most cases were reported to have specific *CXCR4* heterozygous mutations. However, two siblings from Slovenia were found to have an autosomal recessive loss-of-function mutation in *CXCR2* with a frameshift (p.H323fs329X), presenting with myelokathexis and recurrent infections (11). Previous studies have shown that CXCR2 counter-regulates CXCR4 in terms of promoting the release of neutrophils from bone marrow to peripheral blood (12–14). Thus, both loss-of-function *CXCR2* mutations and gain-of-function *CXCR4* mutations cause myelokathexis. Currently, most of the reported cases are in Europe (especially France, Italy) and the United States. In recent years, a few cases have also been reported in Asia (15–17).

To date, all the twelve known heterozygous gain-of-function *CXCR4* WHIM mutations are in the genomic region that encodes the C-terminal domain of the receptor (**Figure 1A**). And c.1000C> T (p. Arg334Ter, also written informally as R334X) is the most frequent (**Figure 1B**), which is consistent with a previous review (18, 19). The nucleotide changes, mutation types, amino acid changes and the proportion of WHIM patients with *CXCR4* mutation are also summarized (**Figure 1B**) (17). Here, we report a child presenting with a combination of WHIM syndrome and Kawasaki disease (KD), and harboring a novel heterozygous mutation in exon 2 of *CXCR4* gene confirmed by clinical targeted sequencing.

## Case Presentation

The boy, born full term, is the first child of Chinese unrelated parents. His mother was healthy during pregnancy. His father had non-cyclic and persistent neutropenia. Besides, he also had lymphopenia, low immunoglobulins and very low circulating B cells. So, he had suffered from frequent respiratory infections, almost once a month, for a long time. But he did not present with monocytopenia or human papillomavirus (HPV) infection. On the third day after birth, the boy was treated for jaundice in a local hospital. Blood routine tests showed decreased white blood cells and neutrophils with no periodic changes, mild anemia, and normal platelet count (**Supplementary Table 1**). After the skin condition was improved, his parents requested discharge with the patient’s leukopenia and neutropenia unsolved. At the age of 1 year, the boy was admitted to our hospital with a history of a recurring fever for six days and cough for two days. Before referral, the boy had a red rash on the torso and was treated with antimicrobials in a local hospital, but without any improvement.

**Figure 2:**
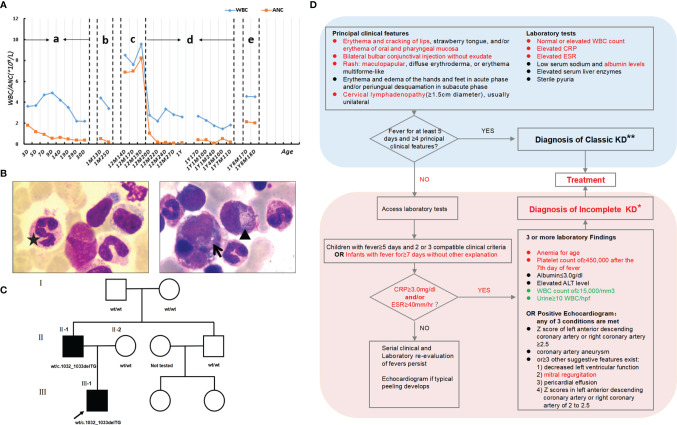
Clinical data. **(A)** Levels of leukocytes and neutrophils of the patient since birth. “a” and “d”: normal stage; “b”, “c” and “e”: stage of infection or immune reaction. **(B)** A bone marrow (BM) smear stained with Wright-Giemsa (×1000) shows a hypercellular BM with mild granulocytic hyperplasia (myeloid: erythroid, 3.19:1) and plentiful granulocytic cells with right-shifted maturation, which is not consistent with peripheral neutropenia. Some neutrophils have coarse granules (▲), cytoplasmic vacuoles (↑), and multi-lobed nuclei (★). **(C)** Pedigree of the family. **(D)** Diagnosis of incomplete KD. Patient clinical features or laboratory tests highlighted in red showed the process of our clinical decision making. Due to WHIM syndrome, laboratory tests highlighted in green are controversial issues in the diagnosis of KD. (_*_) In the absence of a “gold standard” for diagnosis, this algorithm cannot be evidence based but rather represents the informed opinion of the expert committee. Consultation with an expert should be sought any time assistance is needed. (_**_) Patients who lack full clinical features of classic KD are often evaluated for incomplete KD. If coronary artery abnormalities are detected, the diagnosis of KD is considered confirmed in most cases.

Physical examination after admission revealed red maculopapular rashes scattered in the extremities, oral and pharyngeal congestion, red lips, and bilateral congested bulbar conjunctiva without exudate. The patient’s body temperature was 39.5°C and the heart rate was 140-160 bpm with normal heart sounds. Results of lung and neurological examinations were normal. Routine haematologic test results were as follows: white blood cell count (WBC), 9.56×10^9^cells/L; neutrophil count, 8.2×10^9^cells/L; hemoglobin (Hb) level of 103g/L; platelet count, 269×10^9^/L. Laboratory tests revealed a serum C-reactive protein (CRP) level of 133 mg/L, serum procalcitonin (PCT) level of 2.8 ng/mL, and erythrocyte sedimentation rate (ESR) level of 83mm/h. Cellular immune function test revealed CD3+, 70.7%; CD3+CD4+, 62.19%; CD3+CD8+, 7.52%; CD19+, 5.12%; and CD16+56+, 21.25%. Humoral immune function test showed C4 level of 0.115g/L and IgG level of 1.87g/L. Biochemistry test revealed a TP level of 46.8g/L and ALB level of 31.8g/L. Chest X-ray image showed bilateral pneumonia with slight infiltrates in both lungs, which was not typical imaging feature of KD. Based on the above examination results, the boy was initially diagnosed with bronchopneumonia. However, he demonstrated no improvement after being administered potent anti-bacterial treatment and had recurrent fevers over 5 days with a history of a red rash on the torso at the local hospital. So, KD was considered a likely diagnosis, but as insufficient evidence was available to confirm this at the time, a broad-spectrum antibiotic (Latamoxef sodium) was administered empirically. On the next day after admission, echocardiography showed short and thick trunk of the left coronary artery, mild dilated anterior descending artery (internal diameter 2.3mm), and mitral regurgitation. Ultrasonic examination revealed cervical lymphadenopathy (diameter, 14mm). Platelet count increased gradually over normal limits. The above clinical features and laboratory tests were not typical according to the guidelines of the Chinese and American Association (20, 21). However, considering that the boy had granulocytopenia, and his WBC count was rising significantly during the disease course, we thought the boy had been attacked by incomplete KD (iKD) (**Figure 2D**). Upon the diagnosis of iKD, intravenous immunoglobulin (IVIG, 2g/kg), aspirin and dipyridamole were administered, which gradually improved the patient’s condition. On the second day of administration, the boy’s body temperature returned to normal, rash gradually subsided, and heart rate returned to normal. After seven days of IVIG treatment, his lymphadenopathy resolved, serum levels of CRP and PCT were normalized, serum levels of ESR gradually decreased, and platelet count gradually increased (up to 524×10^9^/L), all confirming our clinical decision again. However, his white blood cells and neutrophils in the peripheral blood kept decreasing. Therefore, the patient received bone marrow (BM) morphological examination, which indicated a hypercellular BM with mild granulocytic hyperplasia (myeloid: erythroid, 3.19:1), and plentiful granulocytic cells with right-shifted maturation. Some neutrophils showed coarse granules, cytoplasmic vacuoles, and multi-lobed nuclei (**Figure 2B**). Two weeks after administration, except for leukopenia, neutropenia and low immunoglobulin, his general condition was normal without other specific abnormalities. Then he was discharged and followed up regularly in our hospital. During the follow-up, his cardiac imaging showed that his coronary artery improved without aneurysms or thrombi and platelet count returned to normal. But the trunk at the beginning of the left coronary artery was still short and thick. Besides, his abnormal blood routine and immune function were also unresolved. (**Supplementary Table 2**).

**Figure 3:**
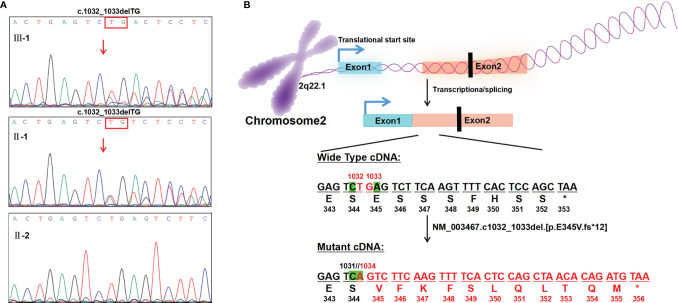
Genetic analysis and illustration of the heterozygous c.1032_1033delTG mutation of *CXCR4* gene. **(A)** Sanger sequencing of *CXCR4* cDNA from the patient and his family, showing TG deletion from exon2(c.1032_1033del)in the proband and his father. The red arrow indicates the variant site of exon2. **(B)** Base deletion (exon2; NM_003467; c.1032_1033delTG) results in original stop-codon loss, frameshift, and prolonged protein.

The patient’s medical history demonstrated that due to leukopenia, neutropenia and low immunoglobulin, he had been suffering from frequent infections, including respiratory tract, gastrointestinal tract and urinary tract infections since birth. Surprisingly, his white blood cell and neutrophil levels could return to normal when he developed infection or inflammation (**Figure 2A**). Blood samples (EDTA, 2ml) from the boy and his parents were collected with their consent and sent to MyGenostics Inc. By high-throughput sequencing in 91 gene exons of granulocyte-related disease, and with the mutation sites with frequency less than 0.05 excluded through 1000 Genomes Project, Exome Variant Server and The Exome Aggregate Consortium database, we found the boy had a novel heterozygous variant in the *CXCR4* gene (NM_003467; exon2) (**Supplementary Table 3**). According to the genetic variation classification criteria and guidelines of the American College of Medical Genetics and Genomics (ACMG) (22), the variation was preliminarily determined to be of unknown clinical significance and the pathogenic grade was PVS1_PM4+PM2+PP3. The variant, c.1032_1033delTG, is a frameshift mutation located in the last 10% of the coding region of *CXCR4* gene, resulting in the original stop-codon loss and the prolonged protein (p. E345Vfs*12), which may make the function of CXCR4 protein changed (PVS1_PM4, **Figure 3B**). The mutation was not found in the normal population database (Exome Sequencing Project EAST, 1000 Genomes Project and The Exome Aggregation Consortium) (PM2). In silico modeling revealed the p.E345Vfs*12 variant is predicated to be damaging or deleterious by predication tools with high confidence, including Mutation Taster (https://www.mutationtaster.org/) and Provean (http://provean.jcvi.org/seq_submit.php) (PP3). Besides, the Combined Annotation-Dependent Depletion (CADD) score (PHRED=34) indicates pathogenicity(https://cadd.gs.washington.edu/score). PCR and Sanger sequencing confirmed the heterozygous variation at this site in the patient’s father, but not in his mother, suggesting that the variation is a novel autosomal dominant mutation (**Figure 3A** and **Supplementary Table 4**). PCR primer pairs were designed by Primer 3.0 online software (http://primer3.ut.ee/). The PCR products were confirmed by Sanger sequencing and analyzed on ABI 3130 Genetic Analyzer (Applied Biosystems).

**Figure 4:**
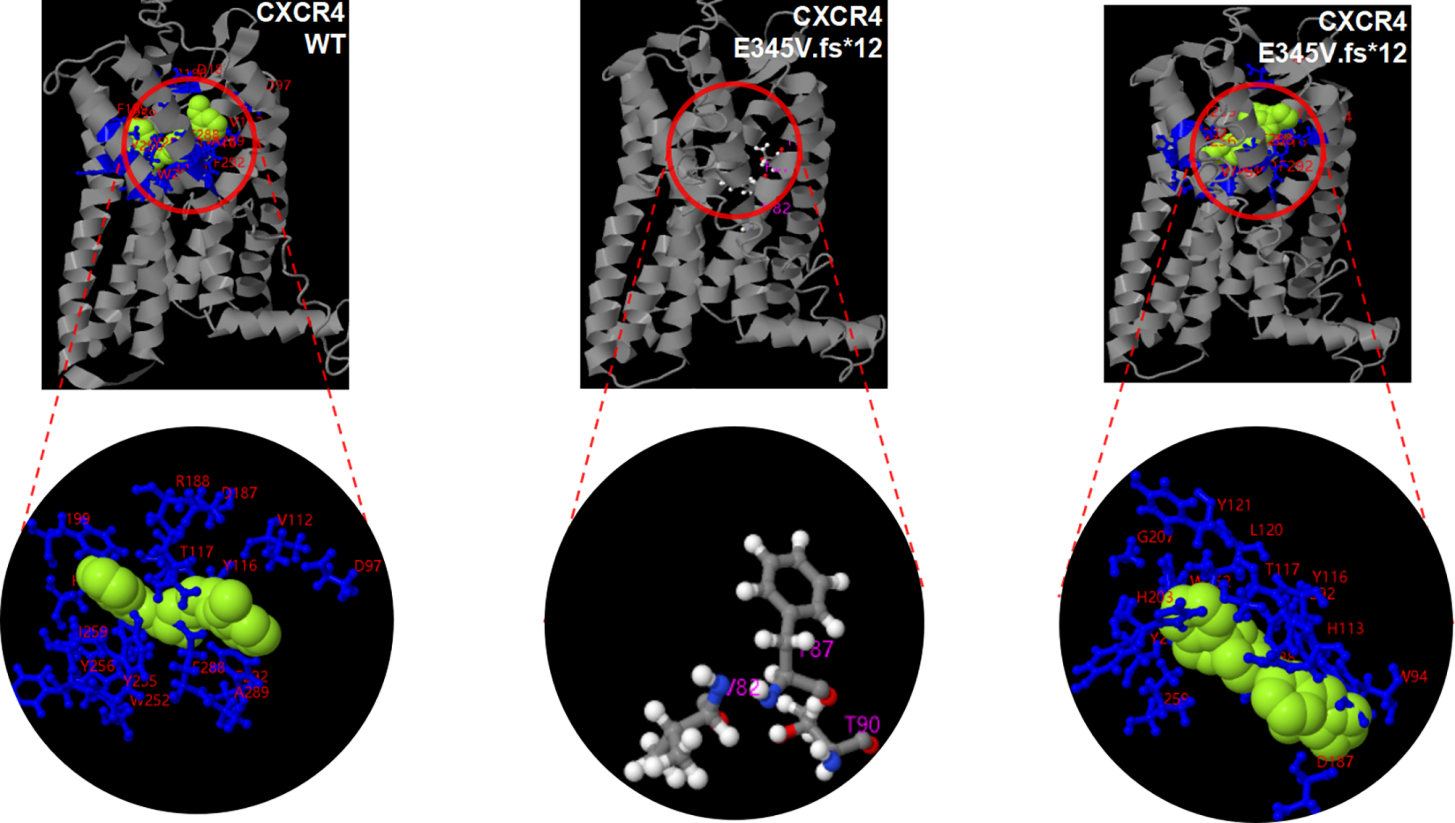
Potentially pathogenic variants may affect the function of CXCR4 protein. 3D modeling of the p. E345V.fs*12 variant reveals disruption of the spatial structure of the N- and C- terminal region and alterations to the position and numbers of ligand binding site residues and active sites, when compared with WT CXCR4. There is no active site in three-dimensional modeling of WT CXCR4 predicted by COFACTOR and COACH. Light green balls represent ligand. Ligand binding site residues are shown by blueline and marked with red number. The active sites are represented by white and gray hockey stick model.

To identify the source of this variation, with the consent of other members of the family, peripheral blood samples were collected from them and sent to Tsingke Biotechnology Co. Ltd. Using the same method, we failed to find this variation in the other family members (**Figure 2C**). Combined with the gene examination results and clinical manifestations, the final diagnosis of WHIM syndrome was made (1, 23, 24). Considering p. E345Vfs*12 is novel mutation, we adopted I-TASSER, ranked as the No. 1 protein structure prediction server in the 14th CASP experiment, to model the mutation and wide type (WT) in the 3D structure of CXCR4 and create PDB files, and used DeepViwer to analyze the molecular structure and graphics (25–27). The results showed that the p. E345Vfs*12 variant significantly disrupted spatial structure of the N- and C- terminal region, when compared with WT CXCR4 (**Figure 4**). Using COFACTOR and COACH based on the I-TASSER structure prediction, the function of WT CXCR4 and the p. E345Vfs*12 variant was further predicted, showing that the ligand binding sites and active site residues of CXCR4 receptors were changed, and the number of them were increased, which may lead to conformational rearrangement (**Figure 4**). We speculate this is the reason for the enhanced stability of p.E345Vfs*12 variant bound to ligands.

At present, the boy and his father have no serious clinical manifestations, except for mild infections. The infections can be treated with antibiotics and well alleviated. Considering chronic sequelae of repeated infection, we strongly recommend them to visit regularly the local hospital for follow-up, and receive immunoglobulin replacement therapy (IgRT) and granulocyte colony-stimulating factor injections (G-CSF) when necessary.

## Discussion

WHIM syndrome caused by *CXCR4* mutation is extremely rare (1). The clinical symptoms are very clear by definition, including warts, hypogammaglobulinemia, recurrent infections and chronic neutropenia, but more than half of the patients fail to show all symptoms, especially young patients who may have warts only or no obvious symptoms (23, 28). To date, exploring the mechanism of combined immunodeficiency in WHIM syndrome still faces great challenges, the reasons for which are as follows: (1) the disease is rare; (2) there is substantial phenotypic heterogeneity; (3) the “WHIM” mouse model cannot fully simulate the human pathophysiology; (4) it is difficult to obtain samples due to leukopenia and neutropenia. Only 5 WHIM-associated *CXCR4* mutations (the NS mutations R334X, G336X, S338X; the MS mutation E343K; the FS mutation L329fs341X) have been studied, whereas signaling properties of remaining mutations are largely unknown (19). It is generally believed that the mutation-caused C-terminal truncation of CXCR4 protein results in decreased phosphorylation, which hinders β-arrestin recruitment and receptor degradation, continuously activating the downstream signals after CXCR4 receptor binds to its ligand CXCL12, then prolonging the residence time of receptors on the cell surface, and thereby resulting in gain-of-function activity and abnormal retention of neutrophils in bone marrow (19, 24). This change accelerates the apoptosis of granulocytes in the bone marrow, leading to cytoplasmic vacuoles and nuclear lysis of granulocytes (1). In addition, the differentiation of immune cells is also affected, leading to mild to moderate hypogammaglobulinemia, which is the main cause of recurrent infection (24). Most reported cases suffer respiratory tract infection, as well as gastrointestinal, skin, and urinary system infection, which can be improved by simple treatment (1). Although the fatality rate of WHIM syndrome is low and the prognosis is good, HPV and Epstein-Barr virus (EBV) infection may develop into tumors, threatening the long-term survival of the patients (18, 29). Some scholars have suggested that when hypogammaglobulinemia complicated with neutropenia occurs, *CXCR4* gene testing should be considered (18, 23). But since *CXCR4* mutations are not found in some families of patients with WHIM syndrome, further exploration is still needed.

The clinical symptoms of our patient are mostly similar to those of WHIM syndrome. The results of gene detection reveal that the boy and his father have c.1032_1033delTG heterozygous mutation, indicating that the variation is inherited from his father. For this variation, there has been no report in the literature database and no pathogenicity analysis in Clinvar database. But this mutation site is in the hot domains of *CXCR4* gene mutation, and there are reports on its upstream frameshift mutation. Moreover, other known variants near the site are closely related to WHIM syndrome (18). We speculate this mutation is highly pathogenic through a variety of prediction software. 3D modeling analysis of the p. E345Vfs*12 variant shows changed spatial structure of the N- and C- terminal region, when compared with WT CXCR4, especially the C-terminal involved in cell signal transduction. In terms of function, the number of ligand binding sites and active sites of CXCR4 protein increases, which may make the binding between receptor and ligand closer, and hinder the internalization of CXCR4 receptor. Besides, after the base deletion (c.1032_1033delTG), the C-terminal amino acid sequence changes significantly. Although the C-terminal amino acid sequence in the cytoplasm is prolonged, the amino acids that can be phosphorylated (such as serine, threonine and tyrosine) are significantly reduced, which may affect the internalization and degradation of CXCR4 receptor. We speculate that this is the root cause of the disease.

Interestingly, WHIM syndrome patients usually appear quite well in between the infectious events. WHIM syndrome is associated with autoimmune diseases, such as diabetes and hypothyroidism (16). To the best of our knowledge, this is the first case of WHIM syndrome with KD, but the nature of the link between these rare conditions is unclear. The most serious outcome of KD is coronary artery disease, which may eventually lead to coronary artery aneurysm or dilatation, ischemic heart disease, or even sudden death. Although the link between these comorbidities and WHIM syndrome is not clear, the key role of CXCR4/CXCL12 signaling axis in autoimmune diabetes, heart and coronary artery development has been demonstrated in mouse models (30, 31). Li et al. have found that the expression of *CXCR4* is strictly regulated in arterial endothelial cells, and the enhanced or down-regulated function of CXCR4 can lead to abnormal development of coronary artery vessels (32). Meanwhile, vascular endothelial cell injury and dysfunction is a pathogenetic mechanism of KD. Some studies have confirmed that the number of endothelial progenitor cells increased significantly in the acute phase of KD (33). Whether *CXCR4* gain-of-function mutation affects or even participates in the progression of KD requires further research. To our surprise, the echocardiographic result reveals short left coronary artery trunk in our patient. Using murine models to investigate the role of CXCL12/CXCR4 signaling in cardiac development, Ivins et al. have found that embryonic Cxcl12-null hearts lacked intra-ventricular coronary arteries and exhibited absent or misplaced coronary artery stems, suggesting CXCL12 is necessary to connect peritruncal and aortic endothelial cells, which is vital in coronary artery formation (31). Hence, we speculate that the novel variation in our patient leads to CXCR4/CXCL12 axis signal disorder and interferes with the development of left coronary artery.

In conclusion, by a clinical sequencing panel, we identified a novel *CXCR4* mutation in a Chinese child with KD manifesting as WHIM syndrome. It should be noted that when a persistent decrease of neutrophils with low immunoglobulin occurs after birth, especially when there is also a family history of neutropenia, immunodeficiency should be investigated in combination with genetic testing.

## Data Availability Statement

The raw data supporting the conclusions of this article will be made available by the authors, without undue reservation.

## Ethics Statement

The studies involving human participants were reviewed and approved by the Ethics Committee of Children’s Hospital of Nanjing Medical University. Written informed consent to participate in this study was provided by the participants’ legal guardian/next of kin. Written informed consent was obtained from the individual(s), and minor(s)’ legal guardian/next of kin, for the publication of any potentially identifiable images or data included in this article.

## Author Contributions

XM, YW, PW, MK, YH, YX, and YF provided clinical information. XM, YW, and PW wrote the manuscript. CC and HL performed the genetic and imaging analysis. YX performed the clinical data analysis. YW, PW, MK, and YF provided critical discussion. XM performed bioinformatic analysis and edited the manuscript. YF supervised the study. All authors contributed to the article and approved the submitted version.

## Funding

This study was supported by the National Natural Science Foundation of China (81903383), Natural Science Foundation of Jiangsu Province (BK20211009), Scientific Research Projects of Jiangsu Health Commission (ZDB2020018), China Postdoctoral Science Foundation funded project (2021M701764), Special Fund for Health Science and Technology Development in Nanjing (JQX19008), Nanjing Medical Science and Technology Development Project (YKK21149), Young Talent Support Project of Children’s Hospital of Nanjing Medical University (TJGC2020016, TJGC2020007, TJGC2020014).

## Conflict of Interest

The authors declare that the research was conducted in the absence of any commercial or financial relationships that could be construed as a potential conflict of interest.

## Publisher’s Note

All claims expressed in this article are solely those of the authors and do not necessarily represent those of their affiliated organizations, or those of the publisher, the editors and the reviewers. Any product that may be evaluated in this article, or claim that may be made by its manufacturer, is not guaranteed or endorsed by the publisher.
