# Supplementary material for: Case Report: A Novel CXCR4 Mutation in a Chinese Child With Kawasaki Disease Causing WHIM Syndrome
Source: Front Immunol. 2022 Apr 13;13:857527. doi: 10.3389/fimmu.2022.857527 (PMC9043559; doi:10.3389/fimmu.2022.857527)
Supplement: Supplementary file 1 [file DataSheet_1.docx]

**Supplementary Table 1:** Changes in blood routine in the patient two weeks after birth

| Age at evaluation | 3d | 5d | 7d | 9d | 14d |
| --- | --- | --- | --- | --- | --- |
| WBC(×10^9^/L)  (Reference) | 3.6↓  （5.0~14.5） | 3.7↓  — | 4.7↓  （5.0~21.0） | 4.9↓  — | 4.2↓  （5.0~20.0） |
| ANC (×10^9^/L)  (Reference) | 1.8↓  （2.0~7.0） | 1.18↓  — | 0.92↓  （1.5~10.0） | 0.539↓  — | 0.64↓  （1.0~9.5）） |
| Lys(×10^9^/L)  (Reference) | 1.32↓  （2.0~5.0） | 1.6↓  — | 2.83↓  （2.0~17.0） | 3.59↓  — | 3.39↓  （2.0~17.0） |
| Hgb(g/L)  (Reference) | 184  （138~218） | 153  — | 140  （140~200） | 131↓  — | 150  （138~198） |
| PLT(×10^9^/L)  (Reference) | 109  （80~320） | 147  — | 167  （100~300） | 228  — | 284  — |

**Supplementary Table 2**: Laboratory results of the patient before and after treatment with IVIG

|  |  | **Pretreatment** | **Posttreatment** |
| --- | --- | --- | --- |
| **Date** |  | **6th September, 2020** | **11th November, 2020** |
| Age at evaluation |  | 12M19D | 1Y1M23D |
| Blood routine |  |  |  |
|  | WBC(×10^9^/L) | 9.56 | 1.76↓ |
|  | ANC (×10^9^/L) | 8.2 | 0.09↓ |
|  | Hgb(g/L) | 103↓ | 120 |
|  | PLT(×10^9^/L) | 269 | 273 |
| Inflammatory markers |  |  |  |
|  | CRP(mg/L) | 133↑ | ND |
|  | ESR(mm/h) | 83↑ | ND |
|  | PCT(ng/ml) | 2.8↑ | ND |
| Immunological markers |  |  |  |
|  | C4（g/L) | 0.115↓ | 0.11↓ |
|  | C3（g/L) | 1.19 | 0.985 |
|  | IgA（g/L) | 0.27 | <0.259↓ |
|  | IgM（g/L) | 0.403 | 0.273↓ |
|  | IgG（g/L) | 1.87↓ | 6.39 |
| Lymphocyte subset quantitation |  |  |  |
|  | CD3+ T cells  (%Lymphocyte, cells/μL) | 70.7, 853.59↓ | 80.82, ND |
|  | CD3+CD8+ T cells  (%Lymphocyte, cells/μL) | 7.52↓, 89.95↓ | 13.27, ND |
|  | CD3+CD4+T cells  (%Lymphocyte, cells/μL) | 62.19↑, 743.92 | 65.47↑,ND |
|  | CD3-CD16+56+NK cells  (%Lymphocyte, cells/μL) | 21.25, 258.91 | 12.1, ND |
|  | CD3-CD19+ B cells  (%Lymphocyte, cells/μL) | 5.12↓, 62.34↓ | 4.65↓,ND |
|  | CD4+/CD8+ cells  (%Lymphocyte) | 0.04 | 0.54 |
|  | CD4+/CD8+ ratio | 8.27↑ | 4.93↑ |
| Biochemistry tests |  |  |  |
|  | ALB（g/L) | 31.8↓ | ND |
|  | TP（g/L) | 46.8↓ | ND |
| **Date** |  | **6th September, 2020** | **4th November 2021** |
| Ultrasonic examination | Herat | The main trunk of the left coronary artery is short and thick; the internal diameter of the anterior descending branch is 2.3mm and that of the circumflex branch is 1.8mm.  mitral regurgitation | The trunk at the beginning of the left coronary artery is short and thick |
|  | Cervical lymph node | Cervical lymph nodes are enlarged, with a maximum diameter of 14mm | ND |
| **Date** |  | **4th September, 2020** | **28th September, 2020** |
| X-ray examination | Lungs | Bilateral pneumonia with slight spotting in both lungs | significant improvement in both lungs |

**Supplementary Table 3**:

Targeted clinical sequencing of 91 genes associated with granulocyte-related diseases

| *ABCD4* | *AGA* | *AP3B1* | *BRCA2* | *BRIP1* | *CASP10* | *CD40* | *CD40LG* |
| --- | --- | --- | --- | --- | --- | --- | --- |
| *CISD2* | *CLPB* | *CSF3R* | *CTLA4* | ***CXCR4*** | *DNM2* | *ELANE* | *ERCC4* |
| *FANCA* | *FANCB* | *FANCC* | *FANCD2* | *FANCE* | *FANCF* | *FANCG* | *FANCI* |
| *FANCL* | *FANCM* | *FAS* | *FASLG* | *FCGR3B* | *FMO3* | *G6PC3* | *GATA1* |
| *GATA2* | *GFI1* | *GRK3* | *GSS* | *HAX1* | *JAGN1* | *KRAS* | *LAMTOR2* |
| *LMBRD1* | *LYST* | *MLPH* | *MMAA* | *MMAB* | *MMACHC* | *MYO5A* | *NRAS* |
| *PALB2* | *PAX5* | *PCCA* | *PCCB* | *PGM3* | *PNP* | *PRF1* | *PRKCD* |
| *PRKCD* | *RAB27A* | *RMRP* | *RPL11* | *RPL15* | *RPL26* | *RPL35A* | *RPL5* |
| *RPS10* | *RPS17* | *RPS19* | *RPS24* | *RPS26* | *RPS29* | *RPS7* | *SBDS* |
| *SBF2* | *SLC35A1* | *SLC37A4* | *SLC46A1* | *SLX4* | *SMARCAL1* | *STK4* | *STX11* |
| *STXBP2* | *TAZ* | *TCIRG1* | *TCN2* | *UBE2T* | *UNC13D* | *USB1* | *VPS13B* |
| *VPS45* | *WAS* | *WFS1* |  |  |  |  |  |

**Supplementary Table 4**:

Information on the novel variant in the *CXCR4* gene of the proband and in silico prediction of disease-causing *CXCR4* heterozygous variant

| Gene | Locus (Hg19) | Transcript | Nucleotide change | MAF  (All) | Provean | Mutation Taster | CADD | Source of variant |
| --- | --- | --- | --- | --- | --- | --- | --- | --- |
|  |  | Exon | Amino acid change |  |  |  |  |  |
| *CXCR4* | chr2:136872464-  136872466 | NM_003467  exon2 | c.1032_1033delTG  p.E345Vfs*12 | **—** | -2.645  Deleterious | Prob:0.999999999986482  Disease causing | PHRED:34 | Father |

MAF, minor allele frequency; Hg19, GRCh37 reference genome;

Red font highlights the p.E345Vfs*12 CXCR4 variant is predicted to be damaging by prediction tools
